# Biophysical and Functional Characterization of a Thermally Stable Bifunctional Serine Protease Inhibitor from *Cleome viscosa* Seeds

**DOI:** 10.3390/ijms262411792

**Published:** 2025-12-05

**Authors:** Manohar Radhakrishnan, Vajravijayan Senthilvadivelu, Eswar Kumar Nadendla, Kundan Sivashanmugan, Gunasekaran Krishnasamy

**Affiliations:** 1 Center of Advanced Study in Crystallography & Biophysics, University of Madras, Chennai 600025, Indianadenlagem@gmail.com (E.K.N.); 2 Department of Biochemistry and Molecular Biology, Indiana University School of Medicine, Indianapolis, IN 46202, USA; 3 Department of Immunology, St Jude Children’s Research Hospital, Memphis, TN 38105, USA; 4 Department of Biochemistry and Molecular Biology, University of Maryland School of Medicine, 655 W. Baltimore St., Baltimore, MD 21201, USA

**Keywords:** *Cleome viscosa*, noncompetitive serine proteases inhibitor, thermally stable protease inhibitor, anti-HSV-2, isothermal titration calorimetry, viral infections

## Abstract

Plant protease inhibitors (PPI) play a significant role against microbes, insects, and, to a considerable extent, human pathogens. PPIs inactivate hydrolase enzymes or depolarize the plasma membrane of the pathogens, thereby inhibiting their growth, replication, and invasion. Here, an active serine protease inhibitor was isolated and purified from the seeds of *Cleome viscosa*. The purified inhibitor was homogenous and exhibited a molecular weight of around 12 kDa as a monomer. The secondary structure analysis indicated that the inhibitor was predominantly composed of α-helical content. The kinetics experiments demonstrated a noncompetitive mode of inhibition towards serine protease when casein was used as the enzyme substrate. The inhibitor formed a stable complex with serine protease, having a likely 1:1 stoichiometry, as inferred from ITC, and the dissociation constant was examined to be K_d_ = 1.9 × 10^−6^ M with a Gibbs free energy of ΔG = −8.079 (kcal/mol). The inhibitor exhibits stable protease inhibition up to 90 °C. Further, in vitro preliminary studies revealed its inhibitory effects against HSV-2 function, evidence that it may have a role in the treatment of viral infections.

## 1. Introduction

Protease inhibitors (PIs) are frequently being investigated for their biological potential and their increasing use in pharmaceutical and biotechnological industries [1,2]. In this context, they have been isolated and characterized from various sources, including bacteria, fungi, protozoa, animals, fish, crabs, and different parts of plants [3]. These small molecular polypeptides are widely present in plants and constitute 10% of plant proteins [4]. In addition, plant protease inhibitors (PPIs) play a potentially defensive role against microbes, insects, and, to a considerable extent, human pathogens [5]. The most extensively characterized PPIs are serpins and cystatins [6]. Among the PPIs, known serine protease inhibitor families are the Bowman–Birk and Kunitz family, barley protease inhibitor family, and potato I inhibitor, potato II inhibitor, and squash inhibitor families [7]. PIs are present in plants and act by inactivating the hydrolase enzymes or depolarization of the plasma membrane of the pathogens, thereby inhibiting their growth and invasion [8]. In addition to their defensive role, their inhibitory properties could be extrapolated to treat several diseases [9]. They also help to regulate function such as apoptosis, cellular signaling, homeostasis, and pathophysiology [10].

Proteases are vital molecules involved in signaling pathways, and any dysregulation in their activity could lead to cardiovascular disease, inflammatory disease, cancer, and neurological disorders. Proteases are critical to the life cycle of many pathogenic viruses, including HIV, hepatitis C [11], Herpes Simplex Virus (HSV) [12] and Human Rhinovirus (HRV). In general, viral proteases cleave large, non-functional polyproteins into smaller, mature proteins essential for viral replication, assembly, and infection. Aprotinin, the known serine protease inhibitor characterized against dengue NS2B-NS3 protease and West Nile virus protease [13]. This makes viral proteases key targets for antiviral drug development. PIs in the form of drugs targeted against these proteases could help in the treatment of the above pathological conditions [14]. Hence, the knowledge of the structure–function relationship becomes inevitable to understand the interaction between the inhibitor and target enzyme in the process of drug designing for several infections [15,16].

**Figure 1:**
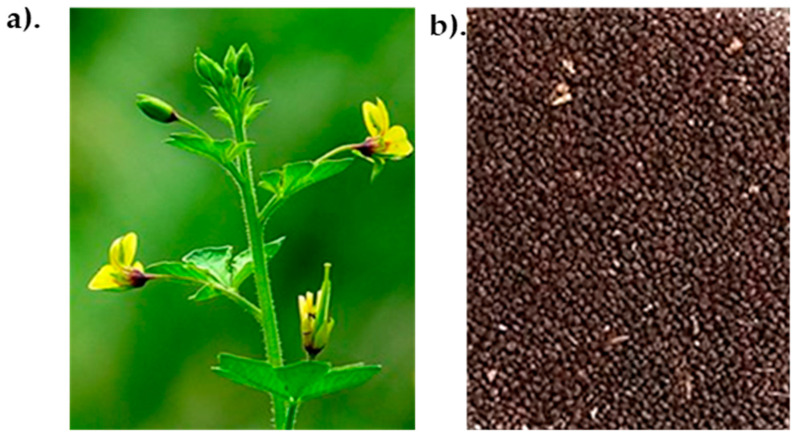
(**a**) *Cleome viscosa* plant, (**b**) seeds used for the study.

Proteases exhibit their substrate hydrolysis action through their catalytic triad residues. In serine proteases, histidine, serine, and aspartic acid are the three key residues within the catalytic pocket [16]. Enzyme inhibition can target the binding site by inhibiting the nucleophilic pocket, resulting in competitive inhibition. Alternatively, the enzyme can be inhibited by binding to a site other than the catalytic pocket, which constitutes noncompetitive inhibition, and this form of inhibition prevents the substrate from being converted into the product. In this study, we have successfully isolated, purified, and characterized a serine protease inhibitor from *Cleome viscosa*, as shown Figure 1a, a medicinal plant belonging to *Brassicaceae* family. Conventionally, *Cleome viscosa* seeds, as shown in Figure 1b, have been used to treat genetic boils in southern India. In addition, various parts of this plant are also used to treat liver diseases, chronic joint pain, and mental disorders.

Its seeds also have been reported to be carminative, anthelmintic, rubefacient, and vesicant [17]. Despite its extensive use in traditional medicine, the mode of action, as well as the biophysical and biochemical properties of its biological macromolecules, remain largely unexplored. Therefore, in the current study, a serine protease inhibitor was purified and characterized to better understand its biological significance. This study provides significant insights into the multi-faceted roles of this serine protease inhibitor in disease treatment and its potential therapeutic applications.

## 2. Results and Discussion

### 2.1. Isolation of the CVTI

Seeds of *Cleome viscosa* were collected from mature plants in Tamilnadu, India, and processed to obtain a crude protein extract. The seeds were initially washed thoroughly to remove surface contaminants, air-dried, and finely powdered. The powdered material was subjected to successive defatting and de-pigmentation treatments using ice-cold acetone and hexane to eliminate lipophilic and pigment components, resulting in a pale, protein-rich seed powder. The crude lysate was prepared by homogenizing the pretreated seed powder in 50 mM Tris buffer (pH 7.8) containing 150 mM NaCl, followed by centrifugation to remove insoluble debris. The supernatant containing soluble proteins represented the crude extract. For partial purification, the crude extract was subjected to stepwise ammonium sulfate precipitation in the ranges of 0–40% and 40–80% saturation. These fractions were then dialyzed extensively against the lysis buffer to remove residual ammonium sulfate, yielding partially purified protein samples suitable for further biophysical and biochemical characterization.

**Figure 2:**
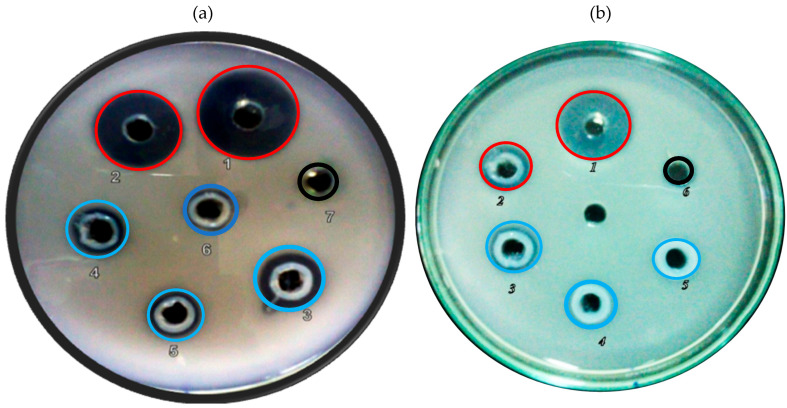
(**a**) Trypsin inhibition activity of *Cleome viscosa* aqueous crude (CVC) extract at pH 7.8; Well No. 1—trypsin 5 µg (positive control), Well No. 2–6—trypsin 5 µg + 10, 20, 30, 40, and 50 µL of CVC, respectively, 7—buffer (negative control). (**b**) Trypsin inhibition activity of 40–80% fraction; Well No: 1—trypsin 5 µg, Well No. 2–5: trypsin 5 µg + 10, 20, 30, and 40 µL of 0–80%, respectively, 6—buffer (negative Control). 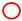
—Trypsin digestion, 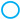
—trypsin inhibition, 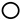
—negative control.

The *C. viscosa* crude (CVC) lysate and 0–40% and 40–80% ammonium sulfate fractions were assayed for trypsin protease inhibition by the agar radial diffusion method, where 1% of casein was used as a substrate. Among the tested samples, interestingly, the CVC and 40–80% ammonium sulfate fraction was shown to have significant protease inhibition activity, which can be seen in Figure 2a,b, respectively, evidencing the trypsin function from the *Cleome viscosa* samples.

### 2.2. CVTI Purification and Trypsin Inhibition

**Figure 3:**
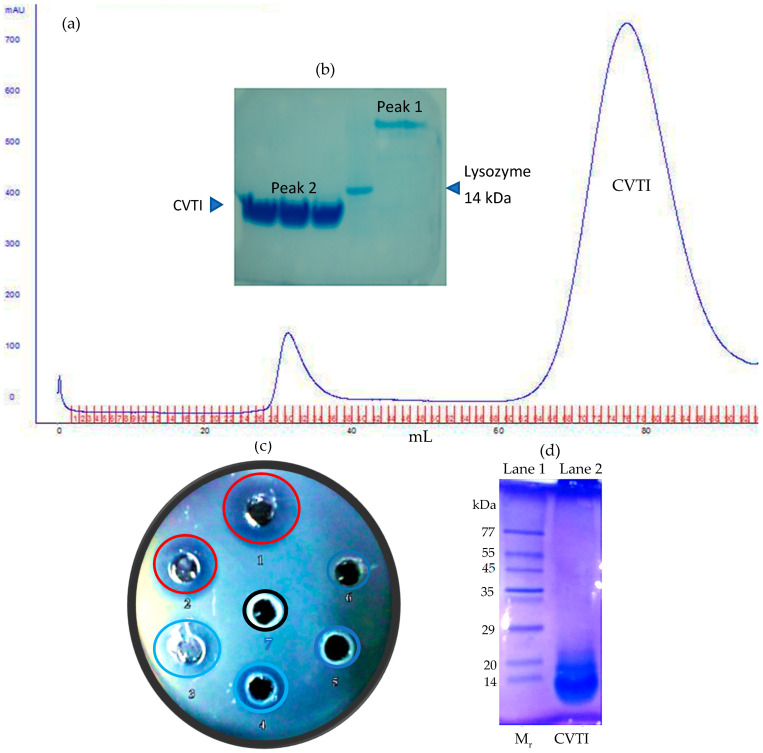
Purification and trypsin inhibition of *Cleome viscosa* trypsin inhibitor CVTI: (**a**) size exclusion chromatography of 40–80% fraction on G-100 resin, (**b**) 15% SDS PAGE with SEC peak 1 and SEC 2. Lysozyme (14 kDa) was loaded as a molecular weight marker. (**c**) Trypsin inhibition by peak 2 fraction of the size exclusion chromatography. (Well No. 1—trypsin 5 µg (positive control), 2—trypsin 5 µg + 10 µg of peak 2 fraction, 3—trypsin 5 µg + 20 µg of peak 2 fraction, 4—trypsin 5 µg + 30 µg of peak 2 fraction, 5—trypsin 5 µg + 40 µg of peak 2 fraction, 6—trypsin 5 µg + 50 µg of peak 2 fraction, 7—buffer (negative control)). (**d**) Purified CVTI with standard protein marker on 12% SDS polyacrylamide gel electrophoresis.

The active functional ammonium sulfate fraction (40–80%) was subjected to a G-100 size exclusion chromatography column, which was equilibrated with SEC buffer (50 mM Tris pH 7.8, 150 mM NaCl), yielding two distinct peaks (Figure 3a), which were subsequently analyzed for purity by 15% SDS PAGE (Figure 3b). Further, peak 2 from the SEC showed significant trypsin inhibition on a 1% casein agar plate (Figure 3c). The inhibition function of all the protease inhibitor samples (CVC, 40–80%, and SEC peak 2) were recorded, measured in millimeter scale and tabulated (Tables S1 and S2). In addition, CVTI also exhibited chymotrypsin inhibition (Figure S1). Together, the electrophoresis and SEC results displayed that the purified trypsin inhibitor was found to be a single polypeptide chain of close to 12 kDa (Figure 3d) as compared to the protein standard molecular weight marker. The inhibitor’s molecular weight correlates with the other plant protease inhibitors from ragi [18], maize [19], velvet bean [20], and corn [21].

### 2.3. CVTI Higher Helical Content Inhibitor

**Figure 4:**
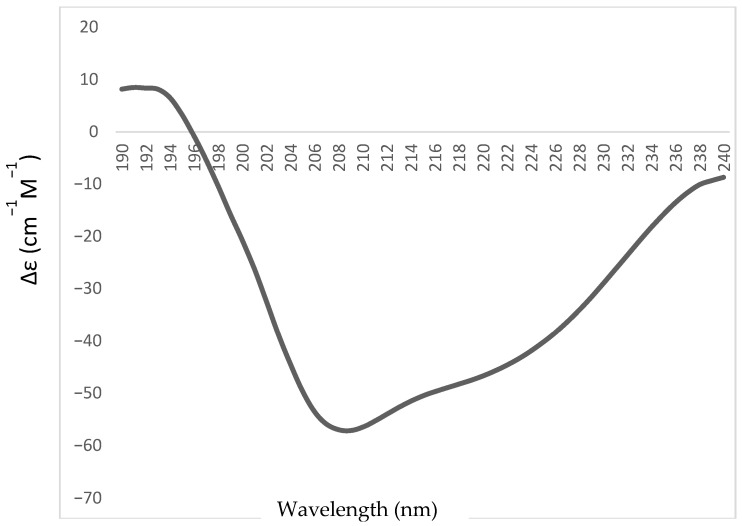
Circular dichroism spectra of CVTI in the far-UV region (190 nm–240 nm), revealing a majority helical content (α-helix: 69.6%).

The secondary structure characterization of purified CVTI was performed using a far-UV CD spectrum (190–240 nm). The negative peak at 210 nm and the broadness of the negative peak up to 220 nm indicate that CVTI is predominantly composed of α-helical content (Figure 4). The maximum positive peak at 190 nm also suggests that the CVTI also contains beta-sheet content. The Dichroweb software predicts that the secondary structure content composition of CVTI has about 69.6% helices, beta-sheets, and others. Hence, CVTI could be grouped into α-helical-type protease inhibitors.

Most of the serine protease inhibitors from plants are beta sheet in nature, such as inhibitors from *Vigna unguiculate* [22] and *Inga cylindrica* [23], or they can be a combination with a high beta content and less alpha content. Few serine protease inhibitors have a majorly helical content. From crystal structure analysis of a 12 kDa, bifunctional amylase/trypsin inhibitor from ragi [18], Hageman factor/amylase trypsin inhibitors from maize seeds were reported to have more than 70% helices [24], and trypsin inhibitors from *Veronica hederifolia* (VHTI) seeds have a helix turn helix binding motif [25]. Hence, the α-helix richness structural characteristics of CVTI may play an important role in serine protease binding.

### 2.4. CVTI Is a Serine Protease Inhibitor

**Figure 5:**
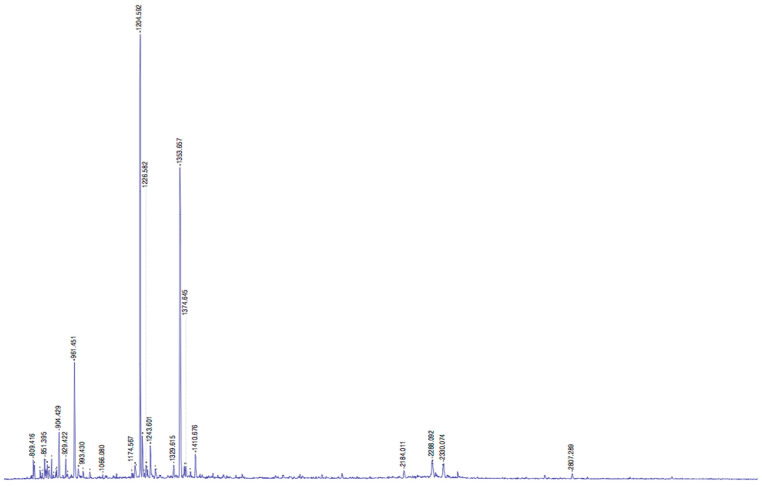
Mass spectrum peptide fingerprinting pattern of CVTI fragments and their corresponding *m*/*z* values.

CVTI was further characterized using mass spectrometry. The *m*/*z* values shown in Figure 5 represent protonated molecular ions; their corresponding peptides were analyzed using mascot server engine peptide mass fingerprinting. From the obtained peptide fragments, the fingerprinting results located four cysteines, as displayed in Table 1. While the top-ranked hit was identified based on *p*-score analysis, it should be noted that the confidence score for this assignment was modest due to insufficient sequence information available for comparison.

The cysteine richness of the inhibitor suggests the potential to form intramolecular disulfide bonds, which is characteristic of many protease inhibitors. The observed structural features probably form disulfide links, which are typical hallmarks of serine protease inhibitors, though these findings warrant careful interpretation given the analytical constraints of confirming the exact sequences from mass-spectroscopy-based measurement. The obtained peptide fragments showed sequence matches with protease inhibitors from various plant sources, including *Triticum urartu*, *Lathyrus sativus*, *Cucumis sativus*, *Solanum phureja*, *Veronica hederifolia*, *Oryza sativa*, *Brassica napus*, and ragi seeds by protein blast analysis, with mass errors of 0.005–0.016% [26]. The sequence alignment of the prominent peptide fragments with related inhibitors is shown in Supplementary Materials Figure S2 using *clustal* omega. While these alignments provide preliminary insights into potential evolutionary relationships and structural conservation among plant-derived protease inhibitors, the interpretations should be considered provisional pending additional validation studies.

### 2.5. CVTI Enzyme Kinetics Studies with Serine Protease V_max_ and K_m_ Determination

**Figure 6:**
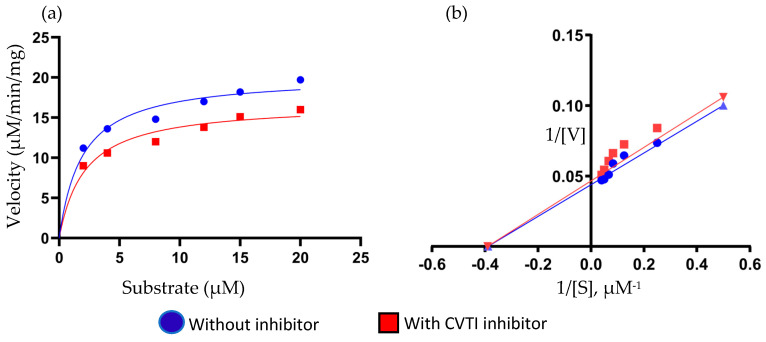
(**a**) Substrate–velocity curve, (**b**) LB plot of CVTI vs. trypsin.

The enzyme kinetics of *Cleome viscosa* trypsin inhibitor (CVTI) toward serine protease trypsin was evaluated using UV-spectrophotometric assays to elucidate its inhibitory mechanism. The substrate–velocity profiles of the trypsin-mediated caseinolytic were determined both in the absence and presence of CVTI (15 µM), as illustrated in the substrate velocity curve (Figure 6a,b). In the uninhibited system, trypsin exhibited a progressive increase in reaction velocity with increasing substrate concentration, ultimately reaching a plateau corresponding to the maximal catalytic rate V_max_ of 20.23 µM/min and K_m_ about 2.1 µM. However, upon the introduction of CVTI, a noticeable decline in V_max_ was observed to 16.7 µM/min and K_m_ to about 1.9 µM. This kinetic behavior strongly indicates that CVTI exerts a noncompetitive mode of inhibition, wherein the inhibitor interacts with the other site distinctly from the active catalytic center. Such binding likely induces conformational alterations in the enzyme structure that reduce its catalytic efficiency without interfering with substrate binding.

Further evidence supporting this inhibitory mechanism was derived from the Lineweaver–Burk (LB) double-reciprocal plot (1/[S]) versus (1/[V]) and, as shown in Figure 6b. The linear plots obtained for both the uninhibited and inhibited reactions intersected on the *x*-axis, reflecting close K_m_ values, while the distinct y intercepts demonstrated a reduced V_max_. This pattern is characteristic of noncompetitive inhibition, confirming that CVTI does not compete with the substrate for the active site but rather modulates enzyme activity through secondary interactions.

Comparable noncompetitive inhibition patterns have been documented for other plant-derived protease inhibitors, including those isolated from *Inga laurina* [27], chickpea (*Cicer arietinum*) [28], and *Dimorphandra mollis* [29]. The similarity in inhibition profiles suggests that such inhibitors may share conserved structural or mechanistic motifs. However, these findings highlight the biochemical significance of CVTI as a potent serine protease inhibitor that employs a classical noncompetitive mechanism. Its high efficacy and stability underscore the evolutionary conservation of plant-derived protease inhibitors as part of natural defense systems and emphasize CVTI’s potential applicability in therapeutic or biotechnological contexts where protease regulation is desired.

### 2.6. CVTI Interaction with Serine Protease

**Figure 7:**
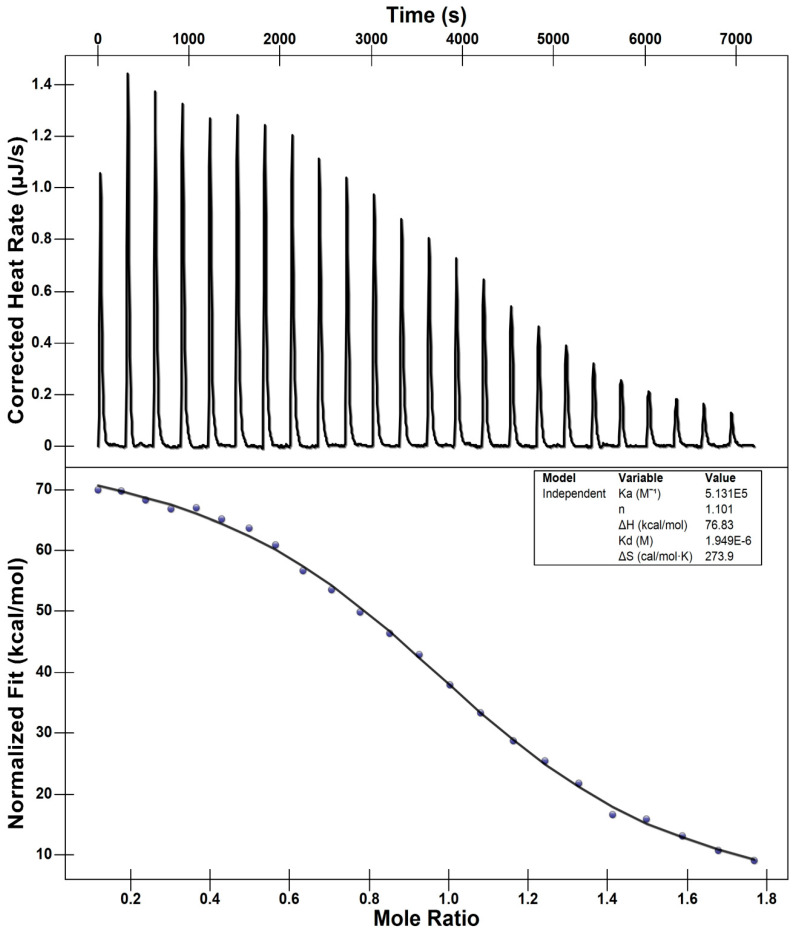
Isothermal titration calorimetry binding interaction of CVTI with trypsin.

Isothermal titration calorimetry (ITC) experiments were performed to characterize the interaction between the serine protease trypsin and the trypsin inhibitor CVTI. The binding thermograms were analyzed using the Nano Analyzer 7.0 software to extract the thermodynamic parameters of the interaction (Figure 7). The analysis revealed a high association constant (K_a_ = 5.13 × 10^5^ M^−1^) and a corresponding dissociation constant (K_d_ = 1.949 × 10^−6^ M), indicating that CVTI exhibits strong affinity toward trypsin and forms a stable inhibitor–enzyme complex [30]. The binding process was characterized by a positive enthalpy change (ΔH = 76.83 kcal mol^−1^) along with a positive entropy contribution (ΔS = 273.9 cal mol^−1^ K^−1^), suggesting that the interaction is predominantly entropy-driven. Such thermodynamic signatures typically indicate the involvement of non-covalent stabilizing forces, including hydrogen bonding and van der Waals interactions, which collectively facilitate proper molecular recognition between the inhibitor and trypsin. The stoichiometry of binding (*n* = 1.101) shows that one molecule of CVTI interacts with one molecule of trypsin, consistent with the 1:1 binding reported for several other plant-derived trypsin inhibitors, such as *Archidendron ellipticum* trypsin inhibitor (AeTI) [31] and mustard trypsin inhibitor (MTI) [32]. This similarity in binding stoichiometry supports the classification of CVTI as a typical canonical trypsin inhibitor. A comparison with previously reported trypsin–inhibitor complexes further reinforces the strength of CVTI binding, and the calculated Gibbs free energy of binding for CVTI (ΔG = –8.079 kcal mol^−1^) is very close to that of white mustard trypsin inhibitor (ΔG = –11.6 kcal mol^−1^), highlighting the energetically favorable nature of the CVTI–trypsin interaction [16].

### 2.7. Thermally Stable Inhibitor

**Figure 8:**
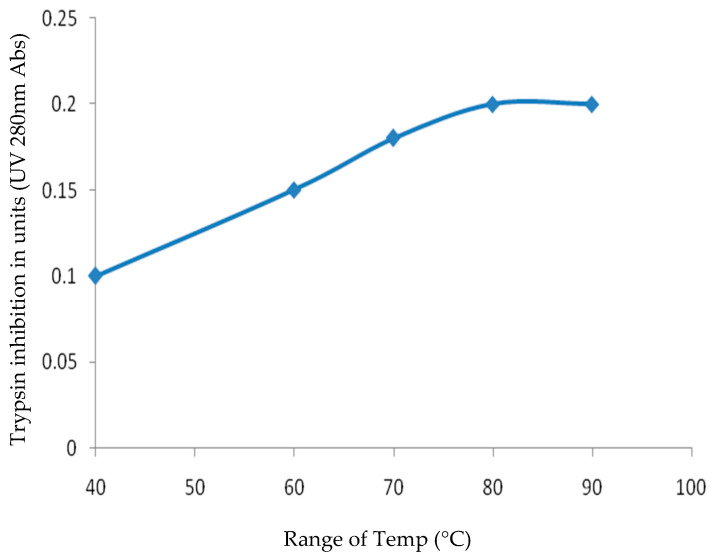
Thermal stability of CVTI.

The trypsin-inhibitory activity of CVTI exhibited remarkable stability across a temperature range of 40 °C to 90 °C at pH 7.8. Notably, UV spectroscopy revealed a gradual increase in activity, particularly between 50 and 70 °C (Figure 8). The retention of inhibitory activity even at 90 °C highlights its exceptional thermal stability, likely attributed to the preservation of the secondary structure that may possibly be due to disulfide linkages formed by cysteine residues. This thermal stability aligns with similar properties observed in trypsin inhibitors isolated from *Sinapis alba* (white mustard) [32] and chymotrypsin/subtilisin inhibitors from *Brassica nigra* (black mustard) [33], the thermal stability of CVTI compared and tabulated with other mustard serine protease inhibitors in Table S3.

### 2.8. Antiviral Role of CVTI Against HSV-2

**Figure 9:**
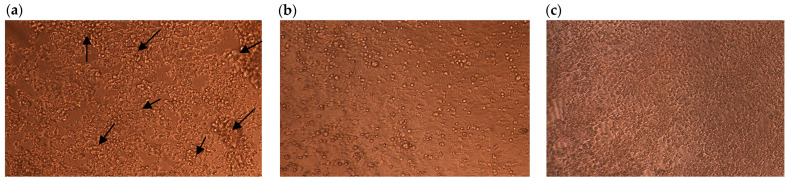
Direct observation of viral infected HEp2 cells under (40×) phase-contrast inverted microscope (arrow indicates the cytopathic effect by viral invasion). (**a**) HSV-2-infected cell; (**b**) acyclovir 1.56 µg/mL; (**c**) CVTI 3 µg/mL.

Several protease inhibitors (PIs) have been reported to exhibit antiviral, anticancer, anti-proliferative, anti-inflammatory, and anti-neurodegenerative properties [34,35,36]. The antiviral activity of CVTI was evaluated in vitro against Herpes Simplex Virus-2 (HSV-2) using HEp2 cells and a controlled viral invasion assay. The cytopathic effect (CPE) caused by HSV-2 on HEp2 cells was observed under an inverted phase-contrast microscope. Severe CPE (Figure 9a) was noted in HSV-2-infected cells, but it was effectively inhibited by acyclovir at 1.56 μg/mL (Figure 9b) and CVTI at 3.12 µg/mL (Figure 9c). The absence of visible CPE confirmed the inhibitory action, with CVTI demonstrating antiviral efficacy comparable to that of acyclovir.

**Figure 10:**
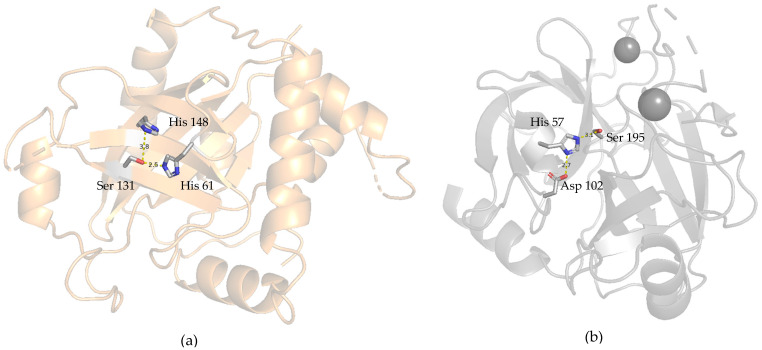
Catalytic site of serine proteases. (**a**) HSV-2 serine protease structure (PDB ID:1AT3). (**b**) Bovine trypsin structure (PDB ID:5JYI).

While many plant-derived serine protease inhibitors are known to have antibacterial, antifungal, and antiviral activities [37,38,39], there are limited reports on their ability to inhibit HSV. This may be due to differences in the catalytic triad of HSV-2 proteases, unlike other trypsin-/chymotrypsin-like serine proteases, where HSV-2 catalytic site residues constitute Ser31/His61/His148 in their active site, compared to the Ser 195/His57/Asp102 configuration in other trypsin-like serine proteases (Figure 10a,b). HSV proteases play a crucial role in DNA packaging, facilitating successful viral replication and invasion. Inhibitors of HSV-1 and HSV-2 have been shown to modulate viral invasion effectively [40].

Our results suggest that CVTI may not directly interact with the catalytic site of the enzyme. Instead, CVTI may exert its inhibitory effect through an alternative mechanism, potentially providing dual inhibitory functionality. Despite structural differences in the catalytic regions among the tested serine proteases, CVTI appears capable of suppressing enzymatic activity by targeting the nucleophilic site or other catalytically essential residues. This alternative mode of interaction may explain its broader inhibitory potential and highlights the need for further structural studies to clarify its mechanism of action. Given the traditional use of *Cleome viscosa* seeds to treat boils, the purified CVTI was investigated for anti-HSV-2 activity and demonstrated significant antiviral properties.

Protease inhibitors in seeds play a critical role in plant survival by protecting seeds from insects and microbes. These seed proteins also provide essential nutrition to animals and humans. Approximately 10% of the soluble proteins in seeds are trypsin inhibitors, which regulate endogenous plant proteases and inhibit gut proteases. In the mustard family, several low-molecular-weight trypsin inhibitors have been identified. For instance, *Sinapis alba* (white mustard) produces single-polypeptide inhibitors with chymotrypsin-inhibitory activity, while *Brassica juncea* (Indian mustard) produces trypsin inhibitors as 2S seed storage proteins. The mustard trypsin inhibitor MT2 has shown anti-insect activity against lepidopteran pests. MT2 expression in *Arabidopsis* was effective against *Plutella xylostella* larvae [41], in *Nicotiana tabacum* (tobacco) against *Spodoptera littoralis* larvae [42], and in oilseeds against *Mamestra brassicae* larvae. Recombinant MT2 expressed in *Pichia pastoris* was also an active inhibitor of *Spodoptera* gut proteases [43].

Additionally, a 14 kDa dimeric molecule consisting of 4 kDa and 9 kDa chains linked by disulfide bonds has been reported in seeds of *Sinapis arvensis* (charlock mustard) and *Brassica nigra* (black mustard). These proteins, part of the serpin family, exhibit bifunctional properties and can inhibit subtilisin and trypsin.

However, no reports of serine protease inhibitors exist for *Cleome viscosa* (wild mustard). Here, we identify a trypsin and chymotrypsin inhibitor from *Cleome viscosa* (CVTI) with dual inhibitory properties, highlighting its potential as a promising candidate for antiviral therapeutics. Though these preliminary findings display CVTI’s dual effect, including serine protease inhibition, and significantly demonstrate preliminary inhibitory activity against HSV-2 function. To elucidate the structural functional relationship the inhibitor and trypsin were complexed and purified by Size exclusion chromatography (Figure S3), the intactness of the complex analyzed by 12% SDS Gel (Figure S4), further structural studies will give insight into the mechanism of CVTI and its mode of action towards these enzymes.

## 3. Materials and Methods

### 3.1. Materials

*Cleome viscosa* seeds were collected directly from fields for isolation. Bovine pancreatic β-trypsin (3× crystallized-salt-free), casein, hen egg-white lysozyme, polyvinyl pyrrolidine, low-range molecular weight marker, and dialysis bags were purchased from Sigma Aldrich (Bengaluru, India) for purification, characterization, and binding studies. Gel filtration resin Sephadex (G100) was purchased from GE Life Sciences (Umea, Sweden). HSV-2 Vero cell [44], acyclovir, minimum essential eagle medium (MEM), Earle’s salts, L-glutamine, sodium bicarbonate, and antibiotic solutions such as penicillin (100 µg/mL), streptomycin (100 µg/mL), kanamycin (50 µg/mL), and amphotericin B (25 µg/mL) were purchased from Sigma Aldrich (Bengaluru, India) for antiviral studies.

### 3.2. Extraction, Isolation of the Inhibitor

Dry seeds of *Cleome viscosa* were collected and thoroughly washed with distilled water to remove dust and then allowed to air dry. Approximately 15 g of seeds was finely powdered and subjected to depigmentation and defatting using 3 volumes of ice-cold acetone followed by 2 volumes of ice-cold hexane. After air-drying, the powder was soaked overnight in 50 mM Tris (pH 7.8 + 150 mM NaCl) containing 1% polyvinyl pyrrolidine at 4 °C to effectively remove phenolic compounds. The suspension was then centrifuged at 12,000 rpm for 30 min at 4 °C to remove debris. The resulting supernatant was incubated at 70 °C for 60 min to deactivate any endogenous protease activity [45]. The clear supernatant, termed *Cleome viscosa* crude (CVC), was collected for further analysis.

### 3.3. Ammonium Sulfate Fractionation

The crude *Cleome viscosa* extract (CVC) was fractionated using ammonium sulfate precipitation. Pulverized solid ammonium sulfate was added gradually to achieve 40% saturation with constant stirring. After standing for 2 h at 4 °C, the mixture was centrifuged at 12,000 rpm for 30 min at 4 °C. The resulting pellet was resuspended in 3 mL of extraction buffer. The supernatant obtained was further subjected to ammonium sulfate precipitation to achieve 40–80% saturation, following the same procedure [46]. These fractions were dialyzed using 10 kDa cutoff dialysis bags against 50 mM Tris, pH 7.8, and 150 mM NaCl buffer at 4 °C overnight, followed by an additional 4 h dialysis with freshly prepared buffer [47].

### 3.4. Trypsin Inhibition Activity

The isolated protein extract crude was assessed for trypsin inhibition activity as described [16,28]. Briefly, one percent (1% *w/v*) casein agar solution adjusted to pH 7.8 was autoclaved and plated, and the wells were made [48]. To 5 μg of bovine pancreatic β-trypsin varying concentrations (using various volumes) of *Cleome viscosa* samples were mixed and incubated at 37 °C for 30 min. The preincubated samples with trypsin were loaded into punched wells along with proper positive and negative controls. Plates were incubated overnight at 37 °C to examine the protease inhibition activity from the digestion of zone in millimeters and tabulated under Supplementary Material section.

### 3.5. Size Exclusion Chromatography

The ammonium sulfate fraction (40–80%) samples that showed anti-trypsin activity were subjected to size exclusion chromatography (SEC) using a Sephadex G-100 column connected to an FPLC (ÄKTA purifier, GE Life Sciences, Umea, Sweden) system. The column was pre-equilibrated with SEC buffer (50 mM Tris pH 7.8, 150 mM NaCl) by 2 column volumes, and fractions were collected at a flow rate of 0.5 mL/min. All the obtained peak fractions at 280 nm were analyzed for their trypsin inhibition activity by the radial diffusion method, as mentioned above. Samples were further analyzed on 15% SDS PAGE to confirm the purity of the sample under denaturing conditions with a standard protein marker. The SDS gel was stained by freshly prepared Coomassie blue R-250 and completely destained to visualize the protein bands [49].

### 3.6. Circular Dichroism Analysis of Cleome Viscosa Trypsin Inhibitor (CVTI)

The far-UV CD measurements of CVTI were carried out using a Jasco J 815 polarimeter with 0.1 mg/mL of CVTI in 50 mM Tris at pH 7.8 + 150 mM NaCl at room temperature. The instrument was calibrated with a standard solution of (+)-10-camphor sulfonic acid. Quartz cuvettes of a 0.1 cm path length (Hellma, Plainview, NY, United States) were used to collect the data at the far-UV (190–240 nm) region with a scanning speed of 50 nm/min. Data were collected as triplets, and the average spectrum was taken for processing after baseline correction with the buffer spectrum. Mean residue ellipticity was calculated and was utilized for secondary structure determination. The Dichroweb software was used to determine the secondary structure content analysis [50].

### 3.7. Protein Identification Using Peptide Mass Fingerprinting

The purified 12 kDa protein was excised from Coomassie blue R-250-stained SDS polyacrylamide gel and digested into fragments by trypsin, as previously mentioned [51]. The trypsinized protein was loaded onto a mass spectrometer (MALDI-TOF/TOF, Bruker, Billerica, MA, USA). The obtained peptide peaks (Bio Tools software, version 2.2 Bruker Daltonics, Billerica, MA, USA) and their corresponding masses were analyzed by the MASCOT search tool, (www.matrixscience.com, accessed on 6 October 2025, Matrix Science Ltd., London, UK). All results were compared to the NCBI database [52].

### 3.8. UV-Based Kinetics

The enzyme–enzyme inhibitor kinetics of CVTI against trypsin were examined using the UV spectroscopic method. Casein was used as the substrate for the kinetics experiment, fixed concentration of CVTI was used with 5 µM trypsin at a fixed reaction time, and the rate of proteolysis was measured and compared in the presence and absence of the inhibitor, as described by [28]. Trypsin (5 µM) with the substrate was taken separately and pre-incubated with CVTI (15 µM) for 20 min at 25 °C in a buffer containing 50 mM Tris at pH 7.8 + 150 mM NaCl. To measure the residual protease activity, substrate of varying concentrations up to 20 µM was taken. The hydrolysis rate was monitored by measuring the peptidyl substrate under UV absorbance at 280 nm [53]. Lineweaver–Burk linear regression plots 1/[S] vs. 1/[V] were obtained with GraphPad Prism 6.0 (GraphPad Software, Inc., San Diego, CA, USA). Assays were carried out in triplicates.

### 3.9. Isothermal Titration Calorimetry Binding Study

A Nano ITC instrument (TA Instruments, Lindon, UT, USA) [14] was used for analyzing the enthalpy and entropy changes resulting from the titration of CVTI with bovine trypsin. All the solutions used were degassed for about 60 min at 270 rpm under a 176 Hg vacuum. A total of 300 µL of buffer (50 mM Tris at pH 7.8 + 150 mM NaCl) was injected into both the cells, and baseline correction was carried out. To confirm the absence of a dilution factor, various concentrations of buffer were used in the protein (trypsin)- and inhibitor (CVTI)-based experiments. A quantity of 2.02 μL of CVTI (200µM) was injected sequentially into a 170 μL titration cell initially containing bovine trypsin (20 µM). A time interval of 250 s was maintained for successive injections of samples. A rotating Hamilton micro-syringe (50 μL) was used to ensure a homogeneous phase via constant stirring of the solution at a speed of 200 rpm [16]. The heat of dilution from the blank titration of ‘buffer to buffer’ was measured, and these heats of dilution were subtracted from the raw data. The results were analyzed using the Nano ITC (7.0) software (TA Instruments, New Castle, DE, USA).

The variation in the Gibbs free energy of mixing was calculated using the following well-known relationship:

ΔG_b_° = −*RT* ln K_b_,

The change in entropy was calculated using the following:

ΔG_b_° = Δ H_b_ − T Δ S_b_.

Data acquisition and analyses were performed using Nano Analyzer, (Nano ITC, TA Instruments, New Castle, DE, USA).

### 3.10. Herpes Simplex Virus-2 Inhibition

HEp2 cells (Sigma Aldrich, Bengaluru, India) were cultured in a 25 cm^2^ tissue culture flask containing minimum essential eagle medium (MEM) supplemented with 10% FBS, Earle′s salts, L-glutamine, sodium bicarbonate, and an antibiotic solution containing penicillin (100 µg/mL), streptomycin (100 µg/mL), kanamycin (50 µg/mL), and amphotericin B (25 µg/mL). Cultured cells were kept at 37 °C in a humidified 5% CO_2_ incubator. The toxicity/viability of HEp2 cells was evaluated by direct observation of treated cells using an inverted phase-contrast microscope. A two-day-old confluent monolayer of HEp2 cells was trypsinized, and the cells were suspended in 10% growth medium. About 100 µL of cell suspension (5 × 10^4^ cells/well) was seeded with HSV-2 cells in a 96-well tissue culture plate and incubated at 37 °C in a humidified 5% CO_2_ incubator. After 24 h, the cells were observed for at least 90% confluency, following which the spent medium was removed from all wells. The test compounds (CVTI, acyclovir) were freshly prepared using 5% MEM to a stock concentration of 1 mg/mL and serially diluted eight times by the two-fold dilution method (100 µg, 50 µg, 25 µg, 12.5 µg, 6.25 µg, 3.125 µg, 1.5625 µg, and 0.78125 µg in 100 µL of 5% MEM). A total of 100 µL of each concentration was added to the respective wells and incubated at 37 °C in a humidified 5% CO_2_ incubator. The plate was observed under an inverted phase-contrast microscope at the 24th and 48th hours; the test wells were compared for cytopathic effects (CPEs) with the drug (CVTI)-treated, untreated (HSV-2), and uninfected cells [18]. The assay was carried out in triplicate.

### 3.11. Thermal Stability of CVTI

Purified CVTI in 50 mM Tris (7.8) and 150 mM NaCl was heated to various temperatures from 40 °C to 90 °C for 20 min and centrifuged at about 8000 rpm at 4 °C. The clear supernatant of 40 µg of CVTI was incubated with 5 µg of bovine trypsin for 30 min at room temperature. The trypsin inhibition activity was then assessed, where 1% *w*/*v* of casein was used as a substrate by a residual caseinolytic, as described earlier in Section 2.8.

## 4. Conclusions

A nearly 12 kDa noncompetitive serine protease inhibitor from *Cleome viscosa* seeds was purified in a three-step process. The negative ΔG value from the ITC results judges the tight binding by following a 1:1 stoichiometry. Enzyme inhibition assays confirmed that CVTI may exhibit a stronger affinity toward trypsin than chymotrypsin, highlighting its potential selectivity within the serine protease family. The high α-helical content of CVTI makes it an α-helix-rich protein, while many other plant serine protease inhibitors fall under the beta case [54]. The correlation of the medicinal importance of the plant with relevance to HSV-2 viral was demonstrated through cell-line-based preliminary anti-HSV-2 assays. Comprehensive structural, mechanistic, and in vivo studies are needed to fully elucidate its mode of action towards these enzymes and validate its therapeutic potential against HSV-2 [55]. CVTI exhibits bifunctional serine protease inhibitory characteristics (Figure S5), as it is capable of inhibiting both trypsin/chymotrypsin and showing antiviral activity against HSV-2. Many of these kinds of protease inhibitors are of a low molecular weight with a flexible structure. However, the crystallization process of such proteins is generally a difficult task and is only possible upon complex formation with a suitable protease, which will stabilize the inhibitor structure. Crystallization efforts of CVTI with trypsin are underway in our lab.

## Figures and Tables

**Table 1:** MALDI-TOF/TOF mass fingerprinting of CVTI sequence fragments.

Peptide	Data Submitted	MH^+^ Matched	Obtained Peptide Sequences
1	2807.387	2807.4566	EEAKKIILKDKPDANIVVL
2	834.3790	834.3905	CVDIRET
3	861.0650	859.4772	CPRILMK
4	2288.02	2289.421	CPRNCDTNIAYSKCPRS
5	120.569	120.461	CLDNCEKEHD

## Data Availability

The original contributions presented in this study are included in the article/Supplementary Material. Further inquiries can be directed to the corresponding authors.

## Supplementary Materials

The following supporting information can be downloaded at: https://www.mdpi.com/article/10.3390/ijms262411792/s1.

## Abbreviations

The following abbreviations are used in this manuscript:

PPI	Plant protease inhibitor
CVC	*Cleome viscosa* crude
CVTI	*Cleome viscosa* trypsin inhibitor
HSV	Herpes simplex virus
CPE	Cytopathic effect
