# Supplementary material for: Biophysical and Functional Characterization of a Thermally Stable Bifunctional Serine Protease Inhibitor from Cleome viscosa Seeds
Source: Int J Mol Sci. 2025 Dec 5;26(24):11792. doi: 10.3390/ijms262411792 (PMC12732657; doi:10.3390/ijms262411792)
Supplement: Supplementary file 1 [file ijms-26-11792-s001.zip › ijms-3943085-supplementary.pdf]

International Journal of *Molecular Sciences*

MDPI

Supplementary material

Table S1. Trypsin inhibition activity of *Cleome Viscosa* crude (CVC) and 40%-80% fraction.

Well no	Sample	CVC Digestion of zone in mm	0%-40% Digestion of zone in mm	40-80% Digestion of zone in mm
1	Trypsin 5µg	21	21	21
2	Trypsin 5µg+10µl	19	21	13
3	Trypsin 5µg+20µl	13	21	12
4	Trypsin 5µg+30µl	12	21	--
5	Trypsin 5µg+40µl	12	21	--
6	Negative control	--	--	--

Table.S2: Trypsin inhibition by Peak 1 and Peak 2 of the size exclusion chromatography.

Well no	Sample	SEC Peak 1 Digestion of zone in mm	SEC Peak 2 Digestion of zone in mm
1	Trypsin 5µg (Positive control)	21	21
2	Trypsin 5µg+10µg	21	19
3	Trypsin 5µg+20µg	21	17
4	Negative control	---	---
5	Trypsin 5µg+30µg	21	--
6	Trypsin 5µg+40µlg	21	--

CVTI inhibits chymotrypsin:

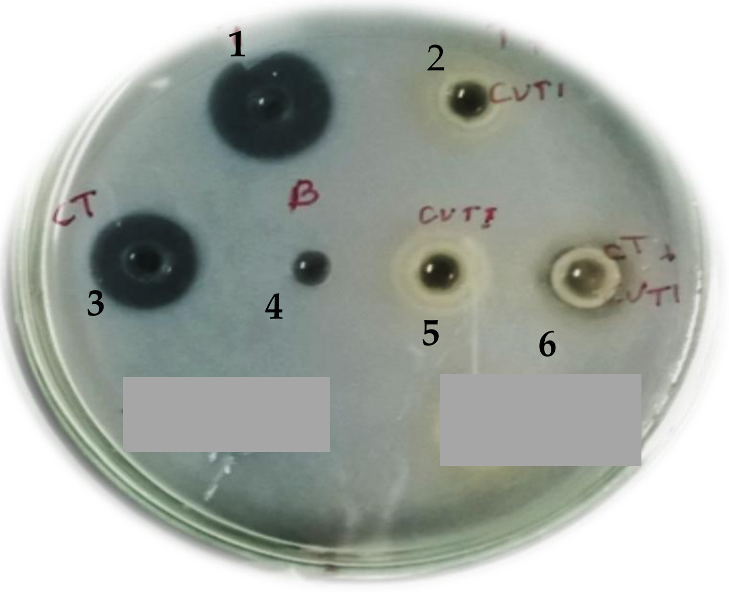

Figure S1. Comparative affinity towards trypsin and chymotrypsin of CVTI: Well No. 1. Trypsin 830 µM, 2. Trypsin 830 µM + *CVTI* 550 µM, 3. Chymotrypsin 800 µM, 4. Buffer 800 µM, 5. CVTI *550* µM, 6. Chymotrypsin 800 µM+CVTI 550 µM.

The affinity of CVTI toward trypsin and chymotrypsin was evaluated using an enzyme inhibition assay, as described in the methods section, with casein serving as the substrate [55]. Comparison of wells 2 and 6 in SI.Fig.1 revealed that chymotrypsin retained a small amount of residual enzymatic activity, whereas trypsin activity was completely inhibited in the presence of CVTI. These findings indicate that CVTI exhibits a stronger inhibitory effect—and therefore CVTI may be higher binding affinity toward trypsin than chymotrypsin. This observation is consistent with previous reports on mustard seed–derived trypsin inhibitors, which have similarly demonstrated a preferential inhibition of trypsin over chymotrypsin.

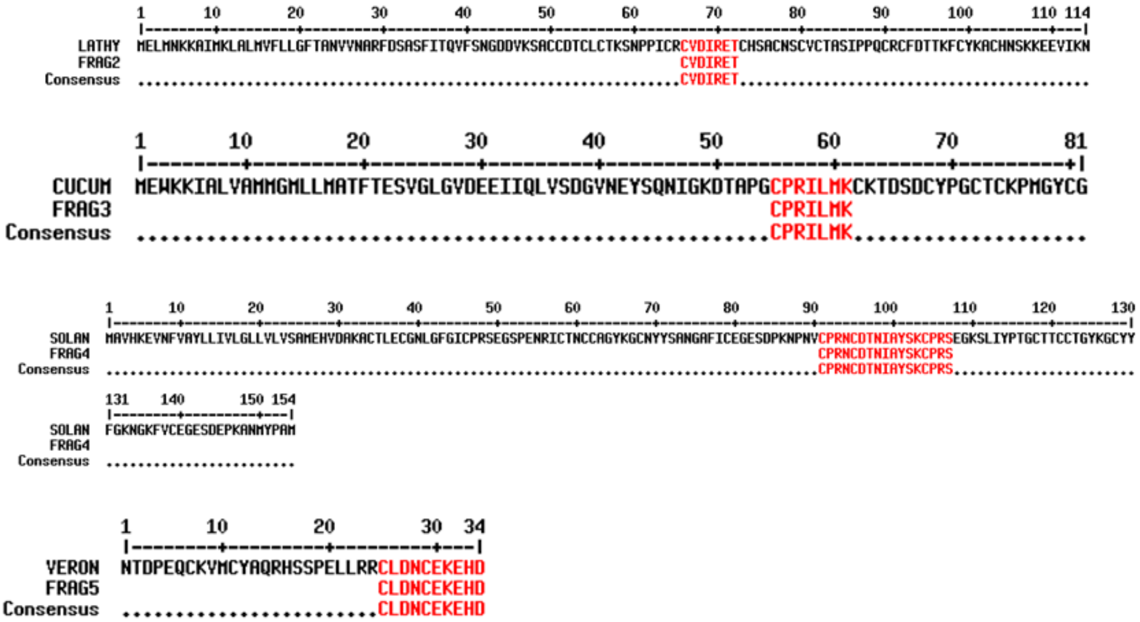

Figure S2. Obtained peptide fragments having cysteine aligned with serine protease inhibitors.

<u>*CVTI* complex with Trypsin:</u>

To investigate complex formation between trypsin and CVTI, the active ammonium sulfate proteins fraction 40%-to 80% were incubated at a molar ratio of 1:1.5, corresponding to approximately 10 mg/mL trypsin and 20 mg/ml of CVTI in 50 mM Tris–HCl buffer containing 150 mM NaCl (pH 7.8). The mixture was allowed to incubate at room temperature (25 °C) for 30 minutes to facilitate protein–protein interaction. Following incubation, the reaction mixture was subjected to separation using a Superdex G75 size-exclusion chromatography column. Fractions were collected at a flow rate of 0.5 mL/min over approximately 1.2 column volumes (SI.Fig.3). The resulting chromatogram displayed two well-resolved major peaks, designated Peak 1 and Peak 2. SDS–PAGE analysis (12% gel) of these fractions, shown in SI. Fig 4, confirmed the identity of the eluted proteins. The band corresponding to Peak 1 (lane 1) showed a molecular mass of approximately 36 kDa, consistent with the expected size of the trypsin–CVTI (enzyme–enzyme inhibitor) complex. Where Peak 2 (lane 3) contained a prominent band at ~12 kDa, matching the molecular mass of free, unbound CVTI. A standard protein ladder was included for reference.

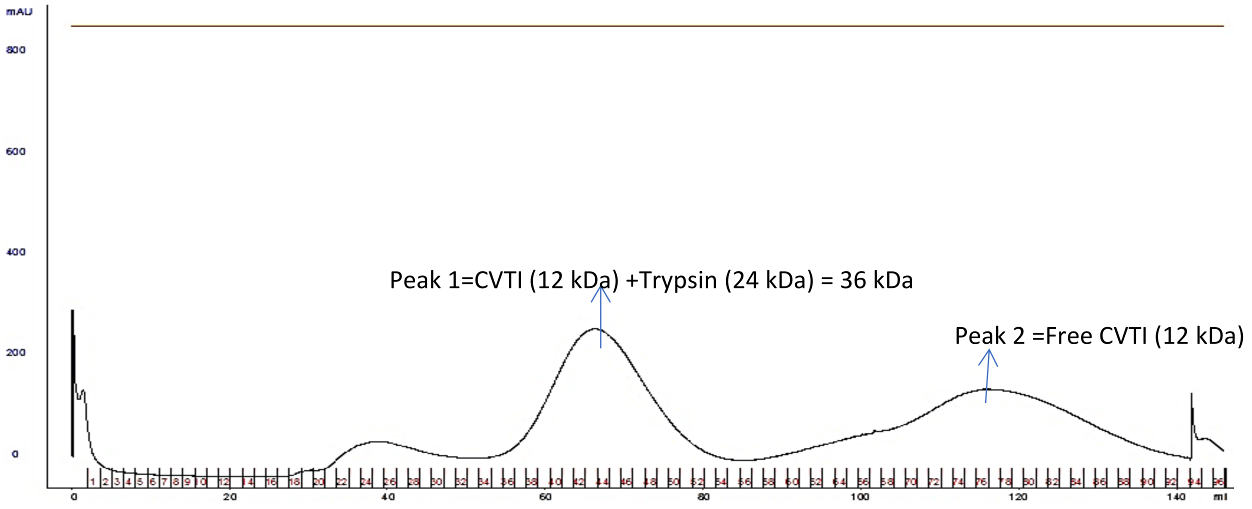

Figure S3. Size exclusion chromatography pattern of CVTI-Trypsin complex (peak 1) and only CVTI (peak 2) on Superdex G-75 column at 0.5 ml/min flow rate. X-axis 0.5ml/min.

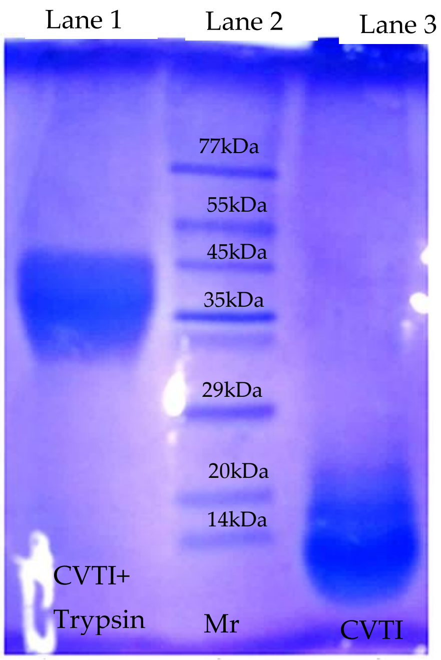

Figure S4: 12% SDS page, 1. CVTI complexed with Trypsin (SEC peak 10, 2. Protein molecular-weight marker. 3.Excess of CVTI (Peak 2 from SEC).

Table S3. Comparison of CVTI with other Mustard Trypsin Inhibitors.

Mustard Name	Molecular weight (Da)	Target enzyme	Temperature stability(°C)
wild mustard (*Cleome viscose*)	12,000	Trypsin, Chymotrypsin	up to 90
White mustard (*synopsis alba*)	18,000	Trypsin	up to 95
Yellow mustard (*Sinapsis arvensis*)	14,000	Trypsin, Chymotrypsin	--
Black mustard (*Brassica nigra*)	15,000	Trypsin, Subtilisin, Chymotrypsin	up to 97
Brown mustard	20,000	Trypsin	--

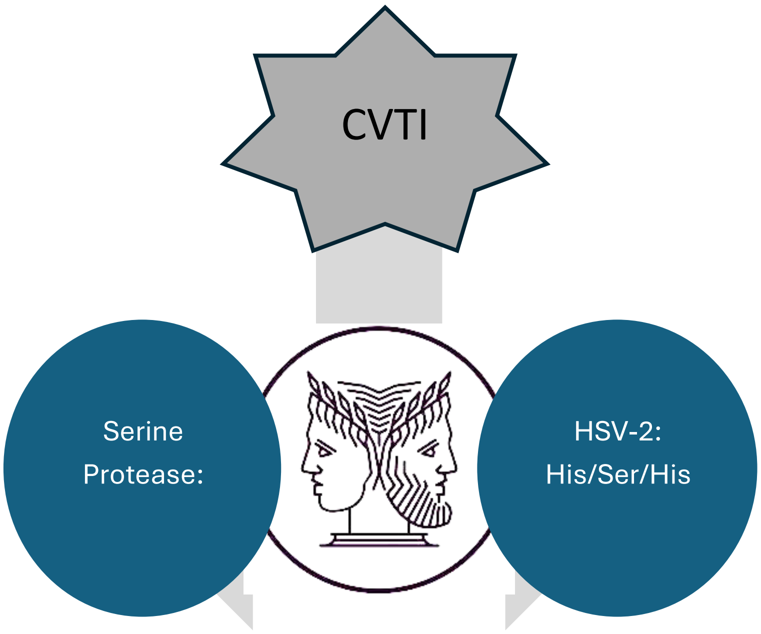

Figure S5. Trypsin/chymotrypsin like serine protease and HSV2 inhibition displays, CVTI an bifunctional inhibitor.
